# Diabetes remains an independent risk factor for adverse remodeling following acute myocardial infarction even with quantification of total infarct size and change in myocardial extracellular volume fraction by CMR

**DOI:** 10.1186/1532-429X-15-S1-P185

**Published:** 2013-01-30

**Authors:** Bobby Heydari, Ravi Shah, Siddique Abbasi, Jiazuo H Feng, Hoshang Farhad, Tomas G Neilan, Ron Blankstein, Rob J van der Geest, Shuaib Abdullah, Sanjeev Francis, Udo Hoffmann, Michael Jerosch-Herold, Raymond Y Kwong

**Affiliations:** 1 Brigham and Women's Hospital, Boston, MA, USA; 2 Department of Radiology, Leiden University Medical Center, Leiden, Netherlands; 3 Massachusetts General Hospital, Boston, MA, USA; 4 North Texas VA Medical Center, Dallas, TX, USA

## Background

Diabetes (DM) has important implications on LV remodeling and prognosis following acute myocardial infarction (MI). We explored the impact of diabetic heart disease independent of traditional risk factors, infarct size, and myocardial extracellular volume fraction (MECVF).

## Methods

We prospectively studied 105 patients with acute MI from the ongoing PROSPECT-CMR clinical trial. Subjects underwent CMR 2 to 4 weeks following MI with follow-up scan at 6-months. Presence of diabetes was determined by history and laboratory testing. Total infarct size was calculated according to previously described full width half-maximum method. MECVF was quantified by serial sampling of T1 ratios of myocardium remote from infarct and blood pool corrected for hematocrit. Sampling was performed in 5-minute intervals beginning 5 minutes and ending 30 minutes after contrast administration from 3 parallel short-axis slices of the heart. Adverse remodeling was defined as a positive change in left ventricular diastolic volume index of greater than 10% at follow-up.

## Results

Thirty-one patients (30%) had a history of type 2 diabetes mellitus (DM) preceding MI. Baseline characteristics and change in mean segmental MECVF are shown in Table 1.

**Table 1:** Baseline Characteristics and Change in MECVF stratified by Presence of DM (n=105)

	DM Present	DM Absent	P value
Age	61.6±9.0	56.8±11.9	0.03
Male	94%	74%	0.03
Hx Hypertension	71%	47%	0.03
Hx Hypercholesterolemia	87%	57%	0.003
Hx Coronary Artery Disease	55%	42%	0.28
BMI	30.4±5.8	28.9±5.4	0.25
LVEF (%)	54.0±10.4	55.0±8.2	0.68
LVEDV (ml)	173.7±50.2	173.0±40.0	0.95
Total Infarct Size (g)	13.6±12.5	15.1±11.3	0.56
Change in mean segmental MECVF	-0.023±0.05	-0.014±0.07	0.53

**Figure 1:**
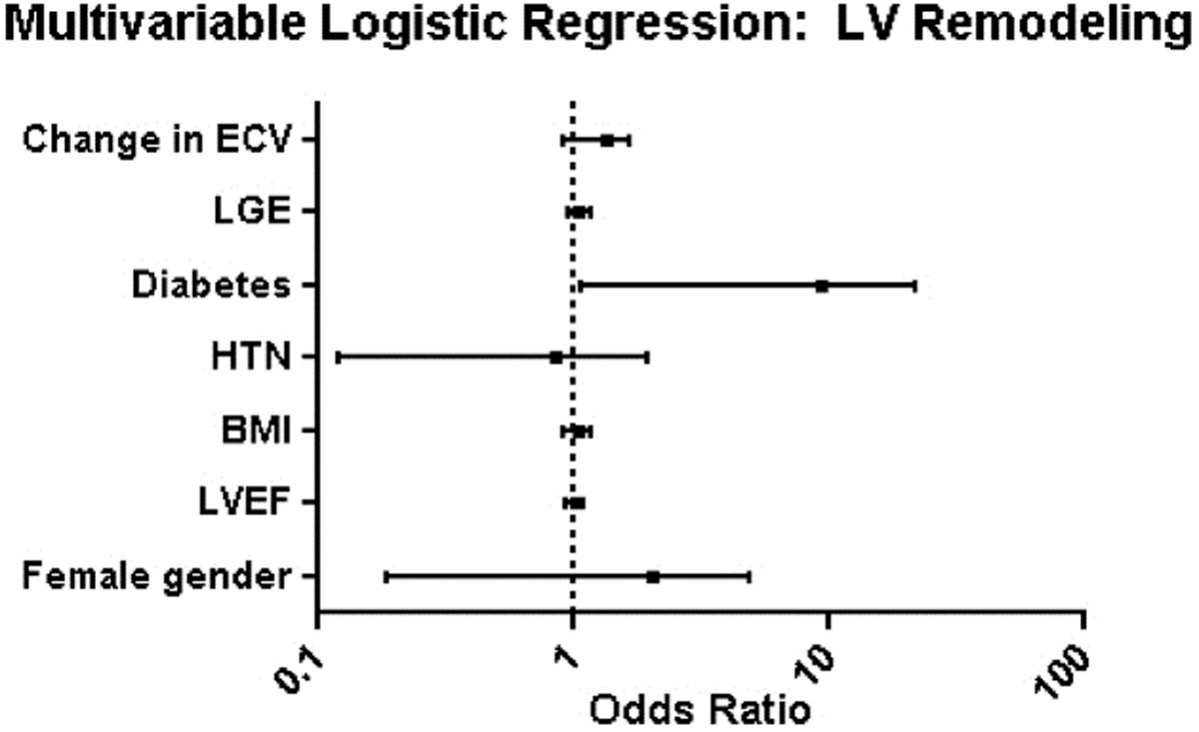


Unadjusted analysis of clinical and CMR characteristics, including change in mean segmental fibrotic index, revealed that only history of DM was associated with adverse remodeling (LR χ2 = 4.6). In multivariable logistic regression analysis, only history of DM remained significantly associated with adverse remodeling at 6 months (LR χ2 = 4.8) (Figure 1). Change in total infarct size, mean segmental MECVF, and other baseline clinical characteristics, including LVEF, did not reach statistical significance.

## Conclusions

Diabetics represent a particularly high risk cohort following acute MI. Our results revealed that presence of DM was an independent risk factor for adverse LV remodeling, including baseline clinical variables, LVEF, change in total infarct size and mean segmental MECVF. We postulate that there are independent mechanisms for adverse remodeling in diabetics, such as genetic predisposition and inflammation, that contribute to adverse remodeling following acute infarction.

## Funding

National Heart Lung and Blood Institute, National Institutes of Health (RO1-HL091157).

Dr. Heydari's salary is supported by the Alberta Heritage Foundation for Medical Research.

